# Supplementary material for: Real-World Dispensing of Buprenorphine in California during Prepandemic and Pandemic Periods
Source: Healthcare (Basel). 2024 Jan 18;12(2):241. doi: 10.3390/healthcare12020241 (PMC10815450; doi:10.3390/healthcare12020241)

Supplementary Figure S1. Trend in monthly volumes of prescribers, patients, and prescriptions in California before and since the COVID-19 pandemic

Note: The intervention, represented by the pandemic, commences at time point 13, indicating the onset of the pandemic during the 13th month of our study window.

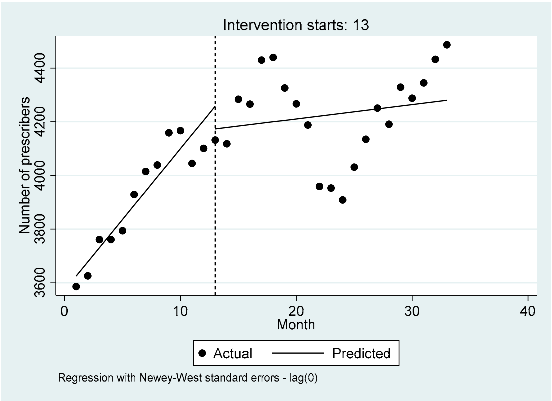

Figure S1. (A) Monthly volume of prescribers.

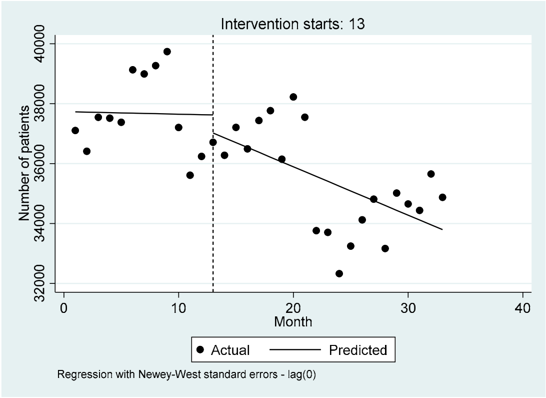

Figure S1. (B) Monthly volume of patients.

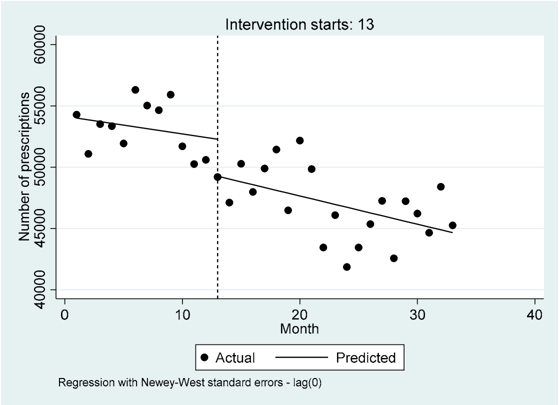

Figure S1. (C) Monthly volume of prescriptions.

Supplementary Figure S2. Trend in average mean daily dosage and mean days of supply for buprenorphine prescriptions in California before and since the COVID-19 pandemic

A Mean daily dosage

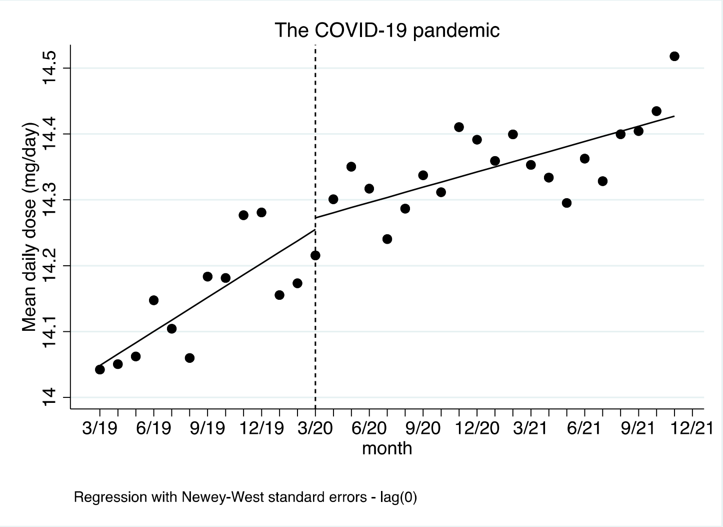

B Mean days of supply

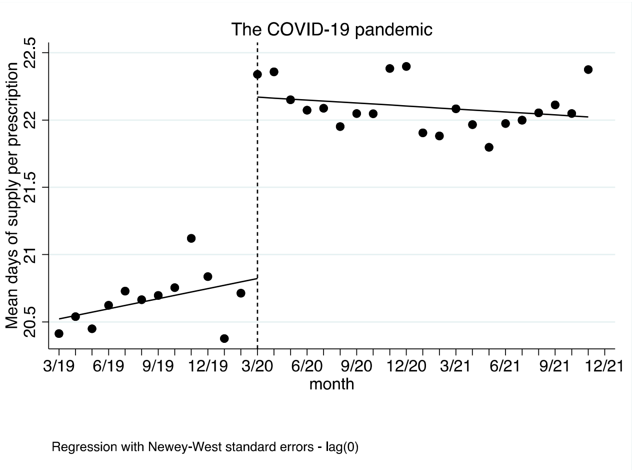

Supplement: Supplementary file 1 [file healthcare-12-00241-s001.zip › healthcare-2747747-supplementary.pdf]
